# Immunomodulatory Effects of Vitamin D in Pregnancy and Beyond

**DOI:** 10.3389/fimmu.2019.02739

**Published:** 2019-11-22

**Authors:** Farhan Cyprian, Eleftheria Lefkou, Katerina Varoudi, Guillermina Girardi

**Affiliations:** ^1^Department of Basic Medical Sciences, College of Medicine, Member of QU Health, Qatar University, Doha, Qatar; ^2^Institute of Obstetric Hematology, Perigenesis, Thessaloniki, Greece

**Keywords:** vitamin D, autoimmunity, antiphospholipid antibodies, pregnancy, placenta, fetal origin of adult disease

## Abstract

In addition to its role in calcium homeostasis and bone formation, a modulatory role of the active form of vitamin D on cells of the immune system, particularly T lymphocytes, has been described. The effects of vitamin D on the production and action of several cytokines has been intensively investigated in recent years. In this connection, deficiency of vitamin D has been associated with several autoimmune diseases, including rheumatoid arthritis (RA), systemic lupus erythematosus (SLE), antiphospholipid syndrome (APS), Hashimoto Thyroiditis (HT), and multiple sclerosis (MS). In a successful pregnancy, the maternal immune response needs to adapt to accommodate the semiallogeneic fetus. Disturbances in maternal tolerance are implicated in infertility and pregnancy complications such as miscarriages (RM) and preeclampsia (PE). It is well-known that a subset of T lymphocytes, regulatory T cells (Tregs) exhibit potent suppressive activity, and have a crucial role in curtailing the destructive response of the immune system during pregnancy, and preventing autoimmune diseases. Interestingly, vitamin D deficiency is common in pregnant women, despite the widespread use of prenatal vitamins, and adverse pregnancy outcomes such as RM, PE, intrauterine growth restriction have been linked to hypovitaminosis D during pregnancy. Research has shown that autoimmune diseases have a significant prevalence within the female population, and women with autoimmune disorders are at higher risk for adverse pregnancy outcomes. Provocatively, dysregulation of T cells plays a crucial role in the pathogenesis of autoimmunity, and adverse pregnancy outcomes where these pathologies are also associated with vitamin D deficiency. This article reviews the immunomodulatory role of vitamin D in autoimmune diseases and pregnancy. In particular, we will describe the role of vitamin D from conception until delivery, including the health of the offspring. This review highlights an observational study where hypovitaminosis D was correlated with decreased fertility, increased disease activity, placental insufficiency, and preeclampsia in women with APS.

## Introduction

**Figure 1:**
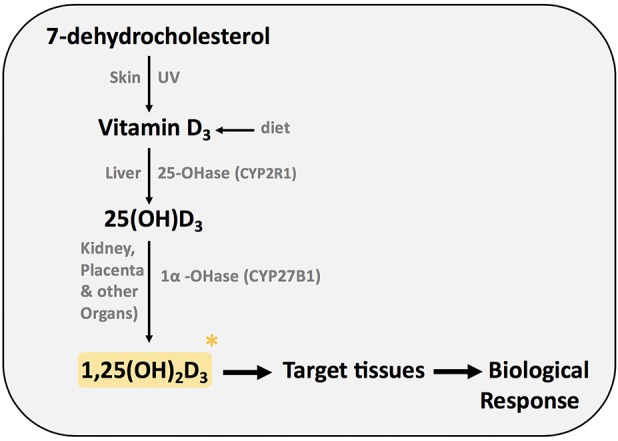
Synthesis of active form of vitamin D (1,25(OH)_2_D_3_).

First, we will briefly summarize the enzymes and precursors involved in the synthesis of the active form of vitamin D (Figure 1). Vitamin D_3_ (cholecalciferol) is taken in the diet or is synthesized in the epidermis from 7-dehydrocholesterol by exposure to ultraviolet irradiation (UV) (1). In order to be biologically active, vitamin D must be converted to its active form.

Vitamin D is transported in the blood by the vitamin D binding protein (DBP). In the liver vitamin D is hydroxylated at C-25 by cytochrome P450 vitamin D 25 hydroxylases, resulting in the formation of 25-hydroxyvitamin D_3_ (25(OH)D_3_). CYP2R1 is the key enzyme required for 25 hydroxylation of vitamin D (1). 25(OH)D_3_ is then hydroxylated in the A ring at carbon 1, resulting in the biological active form of vitamin D, 1,25-dihydroxyvitamin D_3_ (1,25(OH)_2_D_3_). The cytochrome P450 monooxygenase 25(OH)D 1α hydroxylase (CYP27B1; 1α(OH)ase) is present in the kidney and other extrarenal sites such as the placenta, macrophages, lungs, and brain. Despite normal dietary vitamin D intake, mice with mutations in the 1α(OH)ase gene develop vitamin D dependency rickets (VDDR) type 1, highlighting the importance of this enzyme. In this review, we will use the term vitamin D to describe the active molecule 1,25 (OH)_2_D_3_ unless we specify the vitamin D metabolite or precursor particularly investigated.

## Effects of Vitamin D on the Immune System

### Historical Evidence of the Role of Vitamin D on the Immune System

A hundred years ago, the observations of Mellanby suggested a relationship between vitamin D and the immune system. An increase incidence of respiratory infections in rachitic children and dogs was reported in his study (2). Interestingly, vitamin D has been empirically used to treat infections such as tuberculosis (TB) before the discovery of antibiotics. Sunlight exposure and being outdoors was recommended for patients with TB based on the ability of UV to kill bacteria (3). Vitamin D-rich fish liver oil has also been used to treat TB patients. At the time, these observations were attributed to vitamin D deficiency leading to weakness and malnutrition instead of the effect of vitamin D on the immune system. The mechanism of action of vitamin D on the immune system was better understood with the help of molecular biology. We now know that the protective role of vitamin D on the immune system played an important role behind these old therapies to treat TB (4). In this line, present data favor ultraviolet (UV) irradiation and consequent suppression of local and systemic immune responses to reduce the severity of some inflammatory and immune diseases such as psoriasis, multiple sclerosis and asthma (5–7).

Interestingly, recent data demonstrate a link between vitamin D and TB. In this line, patients suffering from TB have shown either vitamin deficiency or vitamin D receptor (VDR) polymorphisms. Furthermore, vitamin D can suppress intracellular growth of *M. tuberculosis in vitro* (8, 9). In addition, the vitamin D-stimulated expression of antimicrobial peptides such as cathelicidin, involved in the first line of defense in TB patients, might be responsible for its protective effect in TB (10).

### Immunoregulatory Effects of Vitamin D

The expression of vitamin D receptor (VDR) in immune cells has highlighted an interesting role of vitamin D in immunity. Today a compelling body of experimental evidence indicates that vitamin D plays a fundamental role in regulating both innate and adaptive immune systems (11). Vitamin D displays a local immune effect via intracellular vitamin D receptors (VDR), that are known to be present in monocytes/macrophages, T cells, B cells, natural killer cells (NK), and dendritic cells (DCs). After binding to its receptor VDR (a member of nuclear receptor superfamily), vitamin D forms a heterodimer with retinoid X receptor (RXR). This complex engages vitamin D Response Element (VDRE) and recruits activators and enzymes with histone acetylation activity. Therefore, the structural changes in chromatin induced by this complex results in the regulation of targeted gene (12).

#### Vitamin D and Innate Immunity

**Figure 2:**
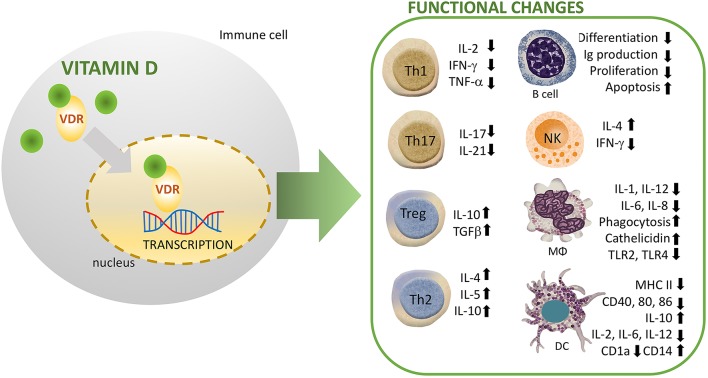
Immunomodulatory effects of vitamin D (1,25(OH)_2_D_3_) on multiple immune cell lineages. NK, natural killer; DC, dendritic cell; R, receptor; VDR, vitamin D Receptor; MΦ, macrophage.

The innate immune system is differentially regulated by vitamin D signaling, where it modulates the synthesis of antimicrobial peptides (AMPs) including, cathelicidin and defensins (13). In this line, promoters of the human genes for cathelicidin, and defensin β2 contain VDRE. NKT cells are thymically derived cells of the innate immune system that produce high amounts of cytokines including IL-4 and IFN-γ. Vitamin D through its interaction with VDR regulates the normal development and function of NKT cells. In this line, NKT cells isolated from VDR knock out mice exhibited diminished secretion of IL-4 and IFN-γ. In addition, vitamin D induced activation in NK cells (14). Recently, Chen et al. studied the effect of vitamin D supplementation on innate immune cells. They observed an enhanced production of IL-1beta and IL-8 by both neutrophils and macrophages, whereas the phagocytic capacity was suppressed in these cells (15) (Figure 2). Other studies have similarly revealed that vitamin D suppresses the activation of macrophages resulting in an anti-inflammatory M2 macrophage phenotype (16). Notably, activation of human monocytes using CD40 ligand and interferon gamma (IFN-γ) have been found to induce VDR and CYP27B1-hydroxylase expression, resulting in enhanced autophagy and antimicrobial peptide synthesis (17). Whereas, vitamin D increases phagocytosis and bactericidal activity of pathogens such as *M. tuberculosis* and *P. aeruginosa* by macrophages (8, 18). Furthermore, the immune-modulating effects of vitamin D and its analogs have been well-characterized in dendritic cells (DCs), which are known to stimulate lymphocytes through antigen presentation. Recent research showed a robust vitamin D-dependent inhibition of maturation, differentiation, and survival of DCs (19). Several *in vitro* and *in vivo* studies have demonstrated a decreased expression level of costimulatory molecules (CD80, CD40, CD86), major histocompatibility complex (MHC) class II, and other maturation-induced surface markers, resulting in impaired maturation of DCs (20) (Figure 2). In response to inflammatory signals, vitamin D strongly impairs the migration and maturation of DCs, which culminates in reduced antigen presentation capacity and activation of T cells. Furthermore, cytokine shift with reduced interleukin-2 (IL-2) production, and increased IL-10 expression, leads to suppression of T helper 1 (Th1) phenotype (Figure 2). Therefore, by maintaining DCs in an immature phenotype, vitamin D and its analogs contribute to an induction of a tolerogenic state (21, 22).

#### Vitamin D and Adaptive Immunity

Early studies have shown that the VDR is highly expressed post-activation in both B and T lymphocytes (23). Among the main functions of vitamin D in the adaptive immune system, the effects of vitamin D on T cells deserve special attention. By binding to the VDR on T cells, vitamin D modulates the differentiation and activation of CD4^+^ lymphocytes (24).

Tregs, a subset of CD4^+^ lymphocytes suppress the immune response and mediate immune tolerance. Several studies have shown that vitamin D promotes proliferation and effector functions of immunosuppressive Foxp3^+^ Tregs (25–27). In humans, vitamin D mediates regulation of suppressive T cells in complicated pregnancies (28). In addition, vitamin D signaling enhances the numbers of Tregs both in patients with inflammatory diseases and healthy controls (29). Interestingly, Vitamin D suppresses T lymphocytes proliferation by reducing IL-2 gene transcription, and inhibiting the production of pro-inflammatory Th-cytokines including, IFN-γ, IL-2, and IL-17 (30) (Figure 2). In agreement with prior studies, immunophenotyping of naïve and memory T lymphocytes in children has revealed an association between vitamin D and risk of infections. In this line, higher vitamin D levels were associated with protection due to increased number of memory T lymphocytes (31). Similarly, a recent study has demonstrated that reduced levels of vitamin D were associated with altered activation of T-lymphocytes in neonates. In particular, measurement of neonates and mothers' cord blood had revealed lower levels of naïve CD4^+^ T cells, CD4^+^ T-helper, and CD8^+^ cytotoxic T lymphocyte in the vitamin D deficient group. In addition, one out of every six infant that presented with sepsis was deficient in vitamin D, suggesting a higher risk of infection in this group (32). Additionally, single-nucleotide polymorphism (SNP) analysis has identified T cell activation RhoGTPase activating protein (TAGAP) and IL-2RA as vitamin D responsive genes of CD4? T cells in patients with multiple sclerosis (33).

It also appears that vitamin D suppresses proliferation and immunoglobulin production in B cells. In addition, it also suppresses the differentiation of B cells into plasma cells (34, 35). Naïve B cells express very low levels of VDR. However, following activation VDR expression in B cells is increased. Vitamin D signaling potentiates apoptosis of activated B cells in presence of relevant stimuli. Moreover, vitamin D inhibits memory B cell formation and secretion of immunoglobulins IgG and IgM in activated B cells (36).

### Vitamin D and Autoimmunity

#### Autoimmunity

In view of the immunomodulatory effects of vitamin D on the adaptive immune response, we will discuss next the significance of vitamin D levels in autoimmune disorders. Autoimmune diseases are characterized by self-tissue destruction via the adaptive immune responses which evade immune regulation. As described above, vitamin D has been defined as a natural immune modulator. Vitamin D regulates the differentiation and activity of CD4^+^ T cells, resulting in a more balanced Th1/Th2 response that limits development of self-reactive T cells preventing inflammation and autoimmunity (37–39). Therefore, a role for vitamin D deficiency in the pathogenesis of autoimmune diseases has been proposed. Several population-based and molecular studies reinforced this observation (34, 40, 41).

As previously described (Figure 2), vitamin D modulates adaptive immune cell functions explaining the significant association between vitamin D deficiency and autoimmune diseases, such as rheumatoid arthritis (RA), systemic lupus erythematosus (SLE), antiphospholipid syndrome (APS), Hashimoto's thyroiditis (HT), and multiple sclerosis (MS) (42–49).

In animal models for MS and SLE, administration of vitamin D either prevented or improved autoimmunity (50). Furthermore, studies performed in mouse models with abrogated vitamin D signaling (dietary or genetic manipulation) demonstrated increased susceptibility to autoimmunity (51–53).

##### Rheumatoid arthritis (RA)

Rheumatoid arthritis (RA) is an autoimmune disorder with a very complex pathophysiology. It is believed to be initiated by a Th1 type response resulting in joint destruction by immune cells (54). The presence of 1α hydroxylase and VDR on macrophages, chondrocytes, and synovial cells in the joints suggest that vitamin D might have a role in RA pathogenesis (55). Accordingly, it has been shown that vitamin D downregulates the production of proinflammatory cytokines IL-1β, IL-6, and TNF-α in macrophages in synovial tissue (42). Therefore, it has been suggested that vitamin D deficiency may increase the risk for the development of RA (56, 57). Consistent with this hypothesis, an inverse correlation between the risk to develop RA and vitamin D levels was described in a large population-based study comprising of almost 30 thousand women (58). Furthermore, evidence continues to accumulate supporting a role of VDR polymorphisms in the pathogenesis of RA (59). *TaqI and FokI* vitamin D receptor polymorphisms have been associated with an increase RA risk (60).

##### Systemic lupus erythematosus (SLE)

Among patients with autoimmune diseases, a higher prevalence of vitamin D deficiency was observed in systemic lupus erythematosus (SLE) (61). Patients with SLE have multiple risk factors for vitamin D deficiency. Increased photosensitivity, responsible for lower sun exposure, leads to a diminished production of vitamin D in the skin. In patients with lupus nephritis, the affected kidney may fail to carry out effective hydroxylation step of 25(OH)D. On the other hand, vitamin D's ability to modulate the immune suggests that hypovitaminosis D might lead to loss of tolerance and production of autoantibodies by B cells (62). In addition, vitamin D insufficient levels exacerbate autoantibody production and disease activity in SLE (63).

##### Antiphospholipid syndrome

Antiphospholipid syndrome (APS) is a systemic autoimmune disease characterized by thrombosis and obstetric complications. Thirty to forty percent of patients with SLE develop antiphospholipid antibodies. These antibodies may activate a cross talk between inflammation and thrombosis leading to adverse clinical events (64). An active crosstalk between inflammation and coagulation involving the complement system and tissue factor (TF), showed to be directly involved in APS-associated pregnancy complications in both mice and women (65–67). Vitamin D has shown not only immunomodulatory but also anti-thrombotic properties. In a study by Agmon-Levine et al., vitamin D deficiency was documented in almost 50% of APS patients vs. one third of controls and was significantly associated with thrombosis (68). In *in vitro* studies, vitamin D inhibited the expression of TF induced by antiphospholipid antibodies. A recent *in vitro* study in vascular smooth muscle cells demonstrated that vitamin D modulates tissue factor and protease-activated receptor 2 (PAR-2) expression (69). Provocatively, TF/PAR-2 signaling has been involved in the pathogenesis of adverse pregnancy outcomes in a murine model of APS (65).

As previously mentioned, complement activation plays a crucial role in adverse pregnancy outcomes in APS in mice and women (70–75). Interestingly vitamin D showed to increase expression of complement inhibitor CD55 (decay accelerating factor) in human monocytes and the associated inhibition of complement activation led to the prevention of preterm birth, adverse pregnancy outcome observed in APS (76). Reinforcing the role of vitamin D in pregnancy in APS, pravastatin that prevented pregnancy complications in APS in mice and women (65, 77) was shown to increase plasma concentrations of 25(OH)D and vitamin D in a rat model (78). Therefore, indicating that vitamin D might also contribute to the protective effects of pravastatin in obstetric APS (OAPS). Vitamin D deficiency is common among APS patients (52) and is also associated with placental dysfunction and adverse pregnancy outcomes (79). Therefore, vitamin D deficiency might contribute to the abnormal placental development and to the adverse pregnancy outcomes observed in OAPS [see Observational Study: Vitamin D levels in Women With Obstetric Antiphospholipid Syndrome (OAPS)].

##### Autoimmune thryroiditis—hashimoto thyroiditis (HT)

Vitamin D serum levels has been associated with the onset and progression of several autoimmune diseases including HT (80). In this line, higher serum 25OHD levels were associated with decreased risk of Hashimoto thyroiditis (HT). In this study, the authors found that vitamin D supplementation leads to a significant decrease in the risk of developing HT (81). Interestingly, a meta-analysis showed a significant correlation between certain VDR gene polymorphisms and HT (82). Animal studies have shown a protective role of vitamin D in the development of experimental autoimmune thyroiditis (83). Vitamin D supplementation, improved inflammation of the thyroid gland by suppressing autoantibodies and proinflammatory cytokines production in mice (83, 84). Interestingly, several studies reported a significant association between vitamin D deficiency with the risk of HT (85, 86). On the other hand, a few studies did not find any link between vitamin D deficiency and the risk of Hashimoto thyroiditis (87, 88).

##### Multiple sclerosis

Multiple sclerosis (MS) is a demyelinating autoimmune condition targeting the central nervous system (CNS) (89). There is a large body of evidence suggesting an association between lack of vitamin D early in life and development of MS (90). Furthermore, a nearly two-fold increased risk of MS was reported in the offspring of mothers that were vitamin D deficient (<30 nmol/L) during early pregnancy (90). Interestingly, UVB-induced vitamin D has shown protective effects in MS patients by upregulating Tregs and tolerogenic DCs (91). Similarly, these effects have also been observed in the experimental autoimmune encephalitis (EAE) mouse model of MS in which vitamin D induces tolerance via Tregs and IDO+ dendritic cells leading to reduced disease severity (92). Notably, vitamin D showed protective effects in a mouse model through the modulation of tight junction proteins in the BBB and nuclear factor kappa B (NFκB) activation (93). The anti-inflammatory effects of vitamin D toward a Th2 immune response may also contribute to its protection of the CNS (37). It is still debatable if the immunomodulatory effects of vitamin D can be used for clinical benefit in MS.

## Role of T Cells in Pregnancy and Its Complications

In the last decade, an integrated mechanism, acknowledging both the innate and adaptive immune systems have been described to explain the maternal immune tolerance required to avoid rejection of the conceptus (94). It is established that during implantation, an active immune suppression is required to prevent an immune response against developing embryo. In this context, Tregs play a central role by repressing cytotoxic T cells, Th1 cells, macrophages, DC and NK cells leading to immune quiescence (95). Hence, both a maternal and fetal immune symbiotic relationship is created to allow a conducive environment for fetal growth and development. Several mechanisms support the maternal immune tolerance at the fetal–maternal interface. First, the adaptive immune response is curtailed by immune suppressive pathways or skewed toward immune tolerance. Second, the immune system contributes to the tissue remodeling necessary for placental development and function. In this context, uterine NK cells (uNK) have a special role facilitating trophoblast migration and the consequent development of the spiral arteries in contrast with peripheral cytotoxic NK cells (96). These uNK cells developed under the influence of IL-15 signaling that is expressed widely in the decidua and placenta (97). Furthermore, macrophages and DCs contribute to the immune tolerance in the gravid uterus. This unique immunological environment, important in maintaining a tolerant maternal–fetal interface is sustained by soluble molecules such as cytokines, chemokines, hormones, and prostaglandins (97). Crucial cell surface proteins, involved in induction of tolerance, are immune checkpoint inhibitors such as programmed death-1 (PD-1) and PD1 ligand (PD-L1). The importance of PD-1/PD-L1 has been demonstrated with an augmentation of this immunosuppressive pathway in normal pregnancies (98). The cytotoxic capacity of CD8^+^ T subset in the decidua is significantly lower compared to peripheral CD8^+^ T cells (99). In line with the importance of immune checkpoint inhibitors in favorable pregnancy outcomes, increased Tregs, have been observed in mouse models (100). In addition to expression of immune checkpoint inhibitors, Tregs secrete immuno-suppressive cytokines TGF-β and IL-10 (101). Complete abrogation of TGFβ signaling leading to Tregs deficiency results in non-viable mice (102). Restoring TGFβ signaling rescues this phenotype (103). In addition, partial TGFβ signaling leads to recurrent pregnancy loss (102). Decidual cytotoxic CD8^+^ T cells are regulated in part via Tregs and relative expression of immune checkpoints (98). The loss of these regulatory mechanisms lead to enhanced CD8^+^ T cell responses and adverse pregnancy outcomes (104).

## The Role of Vitamin D in Pregnancy: From Conception to Parturition

In recent years, “pleiotropic” effects of vitamin D beyond its skeletal regulator functions have been demonstrated. Vitamin D autocrine, paracrine and endocrine functions have been described in numerous organs and systems, in particular the reproductive system. Several studies underscore the role of vitamin D in conception, placentation, progression of pregnancy and pregnancy outcomes including the offspring's health.

Vitamin D deficiency is common in women of reproductive-age (105). In a recent cohort study performed in Norway pregnant women from different ethnic groups showed hypovitaminosis D. Circulating vitamin D levels (<25 nmol/L) were found during pregnancy in women from South Asia (45%), Middle East (40%) and Sub-Saharan Africa (26%) (106). Hypovitaminosis D is a risk factor for infertility and several adverse pregnancy outcomes (107, 108). Furthermore, pre-pregnancy vitamin D levels higher than 75 nmol/L were associated with increased likelihood of pregnancy, reduced pregnancy loss and increased number of livebirths (109).

For many decades it was thought that metabolism of 25(OH)D_3_ only took place in the kidney. However, metabolism of 25(OH)D_3_ was demonstrated in many other organs including the reproductive tract. 25(OH)D_3_ and VDR are present in a variety of female reproductive organs such as pituitary glands, hypothalamus, uterus, oviducts, ovaries, mammary glands, and the placenta (110). In this line, α-hydroxylase expressed in the decidua and placenta highlights the role of vitamin D synthesis in the fetomaternal interface (111, 112). Altogether, vitamin D supports placental development and function by regulating placental calcium transport, and by exerting immunomodulatory effects, critical for pregnancy maintenance (113, 114).

### Role of Vitamin D in Fertility

#### Vitamin D in Female Fertility

Reduced mating success and fertility was observed in female rats with vitamin D deficiency. Vitamin D-deficient diet caused a reduction of up to 70% in the ability to conceive and a significant reduction in the number of viable pups (115). In agreement with the role of vitamin D in mammalian fertility, synergistic effects of vitamin D and progesterone have been observed in ovum implantation in rabbits (116).

While the diminished fertility in mice can be attributed to inadequate calcium levels to induce oocyte maturation, direct effects of vitamin D on the ovaries and hypothalamic–pituitary axis, including brain neurotransmitters such as serotonin, dopamine, and endogenous opioids should be acknowledged (108). In this line, vitamin D biosynthesis and signaling systems were demonstrated in primate ovarian follicles (117). A recent study demonstrated that vitamin D supplementation promoted survival and growth of antral follicles as well as oocyte maturation (117). Correlations between fertility, seasonal variations and geographical regions, have also been observed. It is now clear that these variations are due to changes in vitamin D-levels dependent on UV exposure (118).

Therefore, it is tempting to speculate that vitamin D deficiency might play a role in infertility, a common and distressing issue that affects around 10% of all couples. Consistent with this, a recent systematic review showed an association between serum vitamin D levels and the number of live births in women undergoing assisted reproductive technology (ART) (119). This study suggests that deficiency and insufficiency of vitamin D could be important factors to treat, particularly in women with compromised fertility to improve ART outcomes.

#### Vitamin D and Male Reproductive Physiology

The male reproductive tract is among the widespread systems affected by vitamin D. Expression of VDR, activating enzymes (CYP2R1, CYP27A1, CYP27B1), and inactivating enzymes (CYP24A1) have been demonstrated in the spermatozoa, seminal vesicle, prostate, epididymis including the human testis (120, 121). In addition, vitamin D deficiency has been associated with abnormal spermatogenesis and fertility in animal studies (122). In rats, a significant diminution (73%) was observed in the pregnancy rate when wild type females were mated with diet-induced vitamin D deficient males compared to females mated with vitamin D sufficient-males (122). In support of the observation that vitamin D is required for male fertility, oligoasthenospermia was described in α-hydroxylase CYP27B1 null mice. In this connection, men with vitamin D deficiency also exhibited altered sperm motility (123). Moreover, these α-hydroxylase CYP27B1 null mice also showed hypergonadotrophic hypogonadism suggesting a modulatory role for vitamin D signaling in gonadal function (124). Hypocalcemia has been shown to compromise capacitation and acrosomal reactions, crucial steps in fertilization (125). Therefore, hypocalcemia and hypophosphatemia secondary to vitamin D deficiency may also play an important role in male infertility. Furthermore, diets rich in calcium and phosphorous rescue male fertility in VDR knock-out mice and in male rats on a vitamin D deficient diet (126).

### Role of Vitamin D in Conception

The rapid induction of VDR and α-hydroxylase CYP27B1 in decidua and placenta early in pregnancy highlights a fundamental role of vitamin D in conception, including implantation and the development of the placenta itself (110, 127).

It has been demonstrated that vitamin D binding to VDR upregulates key target genes, such as *HoxA10* crucial for endometrial development, uterine receptivity and implantation (128). The importance of vitamin D in the process of implantation has been further highlighted by studies using knockout mouse models. Both VDR and α-hydroxylase CYP27B1 knock out female mice present with uterine hypoplasia and infertility (129, 130). Injection of vitamin D has been shown to increase uterine weight and promote decidualization of the endometrium in pseudo-pregnant rats suggesting that vitamin D contributes to a crucial step in blastocyst implantation (131).

In addition to its direct role in the decidualization and placentation, vitamin D may also influence implantation and placentation indirectly via its immunomodulatory actions. The immunosuppressive effects of vitamin D during pregnancy and in particular during implantation were postulated many year ago and might contribute to preventing a maternal immune response against the paternal genes-carrying embryo (127). Therefore, throughout pregnancy, decidual synthesis of vitamin D has the potential to modulate uNK cells, DCs, macrophages and T-cells leading to immune tolerance (132, 133). It is well-established that vitamin D inhibits Th1 cytokines while promoting Th2 cytokines, therefore it may favor the process of implantation (133).

### Pregnancy Complications and Vitamin D

#### Preeclampsia (PE)

Fetal cytotrophoblast and differentiated extravillous trophoblasts (EVT) invasion of the maternal decidua and myometrium in the first trimester of pregnancy is key for placentation and successful pregnancy. Interactions between trophoblasts, decidual stromal, and immune cells facilitate implantation and maintenance of pregnancy. Importantly, defective invasion of EVT can cause abnormal placentation and important pregnancy disorders such as miscarriage, PE, intrauterine growth restriction (IUGR), preterm birth (PTB) and stillbirth. Vitamin D deficiency has been associated with increased incidence of pregnancy complications (134). A recent meta-analysis demonstrated an increased risk of PE in women with hypovitaminosis D (135).

Abnormal expression of 1α-hydroxylase has been observed in syncytiotrophoblastic cells from preeclamptic pregnancies (136). Even more, low levels of vitamin D have been found in women that developed severe early onset preeclampsia and vitamin D supplementation showed a protective effect against recurrent PE (137, 138). That PE is characterized by defective placentation at early stages of pregnancy and that hypovitaminosis D is frequently found in women with PE, suggest a potential role for vitamin D as a crucial molecule in normal placentation. An association between VDR *FokI* polymorphism and the risk of PE has also been reported, suggesting that the interaction of vitamin D with its receptor is required for placenta development and function (139). It has also been suggested that low levels of vitamin D may disrupt the immune balance leading to overexpression of Th1 cytokines and failure of immunological tolerance toward embryo implantation (133). In this line, higher expression of Th1 cytokines have been described in placentas of preeclamptic pregnancies, suggesting a protective role of vitamin D at the feto-maternal interface (140).

That both abnormal trophoblast invasion and maternal hypovitaminosis D are associated with abnormal placentation and adverse pregnancy outcomes, suggests a link between vitamin D and EVT migration. Interestingly, vitamin D has been described as a modulator of cellular motility and invasion in cancerous cells (141). In this line, *ex vivo* studies have shown that vitamin D promotes migration and invasion of human EVT isolated from first trimester pregnancies, through enhanced expression of matrix metalloproteinases MMP2 and MMP9 (142).

The molecular mechanisms behind the regulatory effects of vitamin D on cell migration and invasion are not completely understood. Vitamin D has been shown to regulate the actin cytoskeleton in numerous cell types, including trophoblasts (143). In addition, vitamin D restored mobility in umbilical vein endothelial cells (HUVEC) derived from pregnancies affected by PE and gestational diabetes (144). Vitamin D may also exert indirect effects on trophoblast invasion by stimulating the secretion of human chorionic gonadotrophin (hCG) and progesterone (145).

In addition to the detrimental effects in placentation and potential causative effects in PE development, vitamin D deficiency might also contribute to hypertension, a characteristic sign of PE. It is known that suboptimal levels of vitamin D are associated with unfavorable effects on the cardiovascular system. Vitamin D deficiency has been shown to activate the renin-angiotensin-aldosterone system (RAAS) and to induce endothelial dysfunction, both contributing to hypertension (146). In support of the role of vitamin D on the RAAS system, VDR knockout mice displayed disrupted renin expression and angiotensin II production (147). Vitamin D deficiency might also play a role in endothelial dysfunction, a crucial feature in the pathogenesis of PE. 1α-hydroxylase is present in the endothelial and vascular smooth muscle cells protecting the vascular walls through generation of vitamin D (148). Furthermore, vitamin D inhibits endothelial cell activation by cytokines as well as adhesion molecules expression that involves TNF-α (149, 150). Therefore, it has been hypothesized that vitamin D supplementation might help protect endothelial function and control blood pressure in preeclamptic patients (151).

#### Vitamin D in Obesity in Pregnancy

Obesity is a major contributing factor to vitamin D status in pregnancy. While there is no difference between non-obese and obese individuals regarding the synthesis of vitamin D in the skin, the vitamin D concentration in plasma is 57% less in the obese than in the non-obese subjects (152). In this line, it has been demonstrated that excessive adipose tissue causes a decrease in the release of endogenously synthesized vitamin D into the circulation (152). Obesity, a health issue with serious cardiovascular risk also results in higher incidence pregnancy complications associated with increased maternal and fetal morbidity. Studies performed in a large cohort of Chinese couples of reproductive age showed that increases in pre-pregnancy maternal and paternal body mass index (BMI), both independently and combined, increases the risk of adverse pregnancy outcomes such as PTB, low weight birth, and stillbirth (153). The likelihood of conception decreases in a linear fashion with increases in BMI (4% decrease per 1 kg/m^2^ weight gain, starting from a BMI of 29 kg/m^2^) (154). Diminished bioavailability of vitamin D in obese pregnant women leading to reduced immunomodulatory effects at the fetal–maternal interface might explain the adverse pregnancy outcomes in these women. Limited sunlight exposure and nutrient-poor but hypercaloric diets might exacerbate the vitamin D deficiency observed in obese pregnant women, affecting both the mother and the developing fetus. It has been suggested that supplementation with vitamin D might be beneficial in obese patients (155).

#### Preterm Birth (PTB)

PTB, is a major public health concern as it is the main cause of neonatal morbidity and mortality, with an estimated prevalence of 10.6% of live births (156). Epidemiologic studies suggested an association between maternal hypovitaminosis D during pregnancy and PTB (157). It has been suggested that low levels of maternal circulating vitamin D could increase PTB risk and that vitamin D supplementation during pregnancy might help reduce this risk (158).

The onset of labor is caused by an inflammatory response that not only involves the resident immune cells but also the recruitment of inflammatory cells into the reproductive tissues. Of note, there is a significant amount of cytokines/chemokines released at the feto-maternal interface (159). Untimely, premature activation of these inflammatory pathways leads to preterm labor, which can result in PTB. It has been suggested that T cell activation participates in these proinflammatory responses at the fetomaternal interface and cervix during preterm labor. Moreover, T cells with a Th1 phenotype were found in the cord blood of preterm but not in term infants (160). That lower levels of vitamin D are observed in women that delivered preterm, suggests that vitamin D may play an important role in suppressing the maternal immune response and Th1-mediated inflammatory pathways that lead to the onset of labor. In line with vitamin D effects on Tregs, a significant correlation between Tregs and blood vitamin D levels was observed in term and preterm parturition (161). A recent meta-analysis that included 15 trials and 2,833 pregnant women, concluded that supplementation with vitamin D reduced the risk of PTB by 65%, PE by 48% and low birthweight (lower than 2,500 g) by 60% compared with no intervention or placebo (162).

## Maternal Vitamin D Deficiency Association With Fetal Origin of Adult Disease (FOAD)

The surrounding environment affects our health in countless ways. Provocatively, the effects of the environment begin early in life; the maternal womb being the first environment to which the organism is exposed. During the intra-uterine life, the developing fetus is particularly vulnerable to insults, not limited to malnutrition (163).

**Figure 3:**
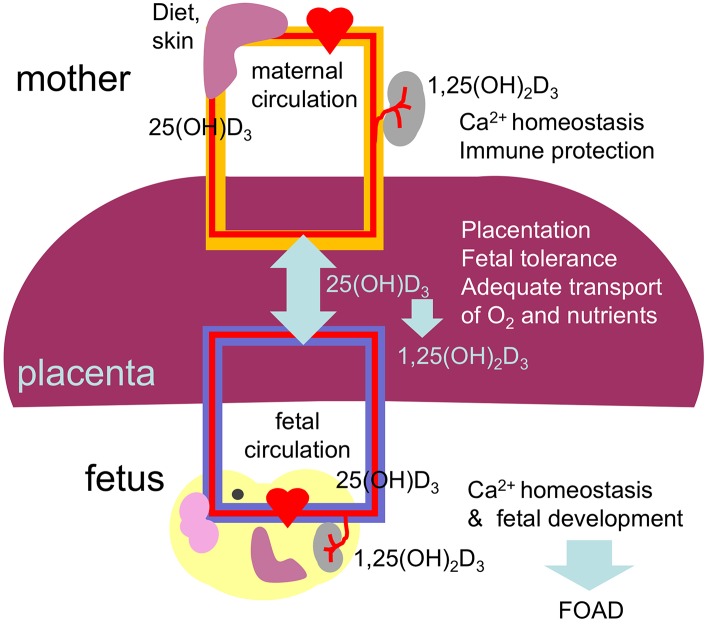
Diagram summarizing the placental transport and role of 25(OH)D3 and 1,25(OH)_2_D_3_ on the placental function and fetal development. Vitamin D during pregnancy is important for placentation (trophoblast migration and remodeling of spiral arteries), immune tolerance, maintaining maternal calcium homeostasis and therefore for fetal development, including the skeletal system and the brain. Low levels of vitamin D during pregnancy can result in abnormal placentation, placental insufficiency and abnormal fetal development leading to compromised health after birth, in agreement with the FOAD concept.

The placenta is formed at gestational week 4 allowing nutrients to reach the developing fetus. From this time until delivery 25(OH)D_3_ easily crosses the placenta reaching concentrations in fetal cord blood equivalent to 87% of the maternal blood levels (164). The biological active vitamin D does not cross the placenta (Figure 3). Interestingly, the placenta and fetal tissues express 1α-hydroxylase leading to bioactive vitamin D in the fetal circulation. Therefore, the fetus depends fully on maternal 25(OH)D_3_ supply and hypovitaminosis D during pregnancy may affect fetal development and future health of the offspring in agreement with the concept of fetal origins of adult disease (FOAD), that Dr. David Barker first popularized (165). The FOAD hypothesis proposes that “events during early development have a profound impact on one's risk for development of future adult disease.” Low birth weight resulting from poor fetal growth and nutrition, is associated to several adult diseases such as, hypertension, obesity, coronary artery disease and insulin resistance (163). It is now well-recognized that the phenotype of an individual can be determined by the nutritional status of the mother. Poor nutrition can lead to hypovitaminosis D. In this context, during “developmental programming” lack of vitamin D during a critical window of development can lead to permanent alterations in physiological processes. In addition, obesity, that is also characterized by diminished vitamin D availability has been associated with adverse health effects not only in the mother but the developing child and offspring later in life (166). Several prospective birth cohort studies followed long-term health outcomes after complicated pregnancies (167).

Epigenetic modification, defined as non-heritable changes in gene expression that are not mediated by alterations in DNA sequence may occur *in utero* (168). *In utero* epigenetic fetal programming may activate specific genes that control fetal development increasing disease risk. Recent studies demonstrated that epigenetic changes of vitamin D catabolism play an important role in increasing vitamin D bioavailability at the fetomaternal interface (169). In addition, it has been shown that maternal vitamin D modifies the expression of the genes encoding placental calcium transporters, influencing bone mineral accrual in the neonate (170). Maternal supplementation with vitamin D during pregnancy significantly reduces the risk of infantile rickets and hypocalcemia (171).

### Vitamin D Deficiency During Pregnancy and the Health of the Offspring—Does Fetal Vitamin D Compromise the Offspring's Immune Function?

#### Vitamin D and Asthma

According to the World Health Organization (WHO), “asthma is the most common chronic disease among children” (172). Several studies demonstrated that prenatal vitamin D status plays a role in the offspring's susceptibility to develop asthma later in life (173, 174). Recent data has suggested a crucial role for vitamin D in reprogramming CD8^+^ T-cells to induce an IL-13-secreting signature, suggesting vitamin D as a promising regulator in asthma (175). That VDRs are present in immune cells and the airways, support this hypothesis (176). Interestingly, polymorphisms in VDR and vitamin D metabolism genes are associated with childhood asthma susceptibility (177).

An association between reduced risk of wheeze in the offspring and high dietary vitamin D intake during pregnancy have been shown by two meta-analyses (178, 179). However, the conclusions of these observational studies are still controversial and randomized control clinical trials are necessary to determine the appropriate levels of vitamin D supplementation during pregnancy on maternal, fetal and perinatal outcomes.

#### Vitamin D, Fetal Neurodevelopment, and Neurocognitive Function

VDR and 1α-hydroxylase have been identified in the fetal brain highlighting the role of vitamin D in brain development (180). In the fetus, serum 25(OH)D_3_ and vitamin D can cross the BBB (Figure 3) bind to VDR and stimulate a wide range of responses, genomic and non-genomic. Furthermore, activated microglial cells *in vitro* have been shown to actively synthesize the active metabolite, 1,25(OH)_2_D_3_ (181). In addition, *in vitro* studies demonstrated that activated microglia increased the expression of the VDR and 1α-hydroxylase, enhancing their responsiveness to vitamin D. Furthermore, activated microglia incubated with vitamin D showed a reduced expression of pro-inflammatory cytokines, IL-6, IL-12, and TNFα, and increased expression of IL-10, indicative of a immunosuppressive effects of vitamin D in the CNS (182).

Vitamin D has also been shown to regulate neurotrophic signaling, including glial derived neurotrophic factor (GDNF) and nerve growth factor (NGF), critical for the survival and migration of developing neurons in the brain (183). Low concentrations of 25(OH)D_3_ during critical stages of development have the potential to affect the reprogramming of the brain tissue structure and function. It has been shown that vitamin D-deficiency leads to fetal mouse brain abnormal morphology and expression of genes related to neuronal survival (184). The ability of vitamin D to regulate neurotrophic factors and modulate inflammation has led to the suggestion that vitamin D is indeed neuroprotective (185). Furthermore, pre-treatment with vitamin D can decrease glutamate-mediated cell death in cultures of cortical, hippocampal and mesencephalic neurons (186). These neuroprotective effects have been recently highlighted in a study showing the inhibitory effect of vitamin D on reactive oxygen species (ROS) toxicity by increasing the synthesis of antioxidant molecules in both glia and neurons (187).

In line with the neuroprotective effects of vitamin D, hypovitaminosis D during the fetal life was associated with greater susceptibility to MS and greater severity of MS symptoms later in life (89). *In vitro* studies in cell culture demonstrated that vitamin D protects neurons from injury induced by modulating T cell activity (188).

On a tissue level, maternal hypovitaminosis D in rats alters the fetal brain morphology leading to psychological disorders in the developing offspring (189). The changes in brain morphology observed in the offspring born to vitamin D-deficient mothers, thinning of neocortex, and ventricle overgrowth, are similar to the ones observed in brains of schizophrenic children suggesting that maternal hypovitaminosis D may be a risk factor for schizophrenia (190). Epidemiological evidence supporting the association between vitamin D exposure in early life and schizophrenia has also been described (191).

Recent studies suggest that maternal vitamin D insufficiency during early pregnancy is also associated with attention-deficit / hyperactive disorder (ADHD)-like symptoms in offspring at age 4 (192). An association between lower first trimester maternal circulating concentration of 25(OH)D_3_ and an increased risk of developing autism in offspring at age 3–7 has been reported in the Chinese population (193). A positive association between lower levels of serum 25(OH)D_3_ (<25 nmol/L) and risk of autism spectrum disorder (ASD) in children was also described in studies performed in Sweden and Iran (194, 195). Moreover, a recent study demonstrated a correlation between mid-gestation vitamin D deficiency and the risk of developing clinical ASD with severe intellectual impairment (196). While results from the latest epidemiological studies support the concept that prenatal vitamin D status impacts the neuropsychological development of children, further research is needed to confirm these observations.

Taking into consideration the important protective effects of vitamin D in fetal development and the future health of the offspring, screening of vitamin D levels during the preconception period and the first trimester of pregnancy should be recommended in women with high risk of hypovitaminosis D, such as women with high body max index, dark skin or autoimmune diseases in order to implement appropriate treatment to prevent adverse pregnancy outcomes, fetal developmental abnormalities and future compromise of the offspring's health in general.

## Observational Study: Vitamin D Levels in Women With Obstetric Antiphospholipid Syndrome (OAPS)

The association between hypovitaminosis D and dysregulation of the immune system, in particular T cells, leading to autoimmunity and adverse pregnancy outcomes prompted us to investigate the levels of vitamin D in women with OAPS. Knowing that T cells play a crucial role in conception and maintenance of pregnancy, we hypothesized that there is a correlation between vitamin D levels and fertility and pregnancy outcomes in women with OAPS. We also determined the association between vitamin D levels and markers of disease activity (presence of flares and complement C3 consumption) in women with OAPS. Co-presence of autoimmune disease Hashimoto thyroiditis (HT) was also investigated.

### Patients and Laboratories Determinations

This observational study, was performed at the Perigenesis, Institute of Obstetric Hematology, Thessaloniki, Greece. All studies in women were performed in strict agreement with the Greece National Bioethics Commission. All patients gave written informed consent.

Antiphospholipid syndrome (APS) was defined by the presence of clinical and laboratory criteria described in the “International consensus statement on an update of the classification criteria for definite antiphospholipid syndrome” (APS) (197). Clinical criteria included one or more clinical episodes of thrombosis and pregnancy morbidity. Pregnancy morbidity was defined as one or more unexplained fetal deaths at or beyond the 10th week of gestation or one or more premature births before the 34th week of gestation due to placental insufficiency such as PE or three or more unexplained consecutive spontaneous abortions before the 10th week of gestation. The laboratory criteria for APS includes the presence of lupus anticoagulant (LA) and/or anticardiolipin (aCL) and/or β2 glycoprotein-I IgG or IgM antibody in plasma or serum on two or more occasions, at least 12 weeks apart (197).

Seventy-six women met the criteria for OAPS before the current pregnancy. All women received conventional low dose aspirin plus low molecular weight heparin (LDA+LMWH) treatment since the beginning of pregnancy (198). Median age for the patients was 37.5 years (IQR 33–40.5).

Vitamin D levels were measured in all women during the first trimester of pregnancy using ELISA tests. Testing for vitamin D is part of standard patient care in Greece. Vitamin D levels were classified as normal (>30 ng/mL) and hypovitaminosis D (<30 ng/mL). Hypovitaminosis D was further classified as deficiency (20.1–29.9 ng/mL) and insufficiency (<20 ng/mL). None of the 76 women received vitamin D supplementation.

Complement activation plays a key role in the pathophysiology of OAPS (70–75). In addition, measuring complement C3 serum levels is a routine practice to monitor disease activity in patients with autoimmune diseases. Therefore, levels of C3 in OAPS women were determined by ELISA. The values of complement component C3 were grouped in three categories (normal: 60–150 mg/dl, low and high). All laboratory tests were performed in the first trimester of the current pregnancy.

Pregnancy complications were classified as follows:

Preeclampsia (PE) was classically defined as a systemic syndrome characterized by new-onset of hypertension and proteinuria in pregnancy. Early onset PE was defined as PE that develops before 34 weeks of gestation. Preterm birth was defined as any birth before 37 completed weeks of gestation. Placental insufficiency refers to placental dysfunction characterized by increased resistance of uteroplacental blood vessels resulting in increased uterine arteries pulsatility index (>95thcentile).

Implantation failure was defined as the inability to achieve a clinical pregnancy after transfer of at least four good-quality embryos in a minimum of three fresh or frozen cycles.

Flares were defined as the relapse of symptoms that can compromise the skin, the joints, or any other compromised organ.

Hashimoto's disease diagnosis was based on blood tests showing lower serum T3 (triiodothyronine) and T4 (thyroxine) levels (<10% of the reference values) with normal thyroid-stimulating hormone levels and the presence of antithyroid antibodies [anti-TPO (anti-thyroid peroxidase) and anti-Tg (anti-thyroglobulin) antibodies].

### Statistical Analysis

All analysis were conducted with GraphPad Prism statistical software (GraphPad Software Inc.). Absolute and relative frequencies were calculated. Fisher's exact test was performed. Two tailed *p*-values were calculated. *P* <0.05 was considered statistically significant.

#### Exploratory Data Analysis

Data from seventy-six pregnant women with OAPS were analyzed. In agreement with the literature (45, 84, 199, 200), a high percentage (77.6%) of these patients showed hypovitaminosis D (Table 1). Within this group, 64.4% of the women were vitamin D deficient and 35.6% vitamin D insufficient in the first trimester of pregnancy. Only 17 out of the 76 patients (22.4%) showed vitamin D levels within the normal range. Of note, wearing sunscreen, limited exposure to sun light, dark skin and dairy products not supplemented with vitamin D in Greece might contribute to hypovitaminosis D in this geographic area.

**Table 1:** Vitamin D levels, fertility and pregnancy outcomes, complement levels, disease activity and co-presence of Hashimoto Thyroiditis in OAPS patients.

	**OAPS patients (*****N*** **=** **76)**		
**Vitamin D levels**	**Normal *N* = 17 (24.3%)**	**Hypovitaminosis D** ***N*** **=** **59 (77.6%)**	
		**Deficiency**	**Insufficiency**
		***N*** **=** **38 (64.4%)**	***N*** **=** **21 (35.6%)**
Implantation failure	1 (5.9%)	8 (21%) *p* = 0.2469	6 (28.6%) *p* = 0.2204
		14 (23.7%), *p* = 0.1669	
IVF	2 (11.8%)	15 (39.5%) *p* = 0.0584	12 (57.1%)^*^p = 0.0063
		27 (45.8%)^*^, *p* = 0.0116	
Low C3 levels	1 (5.8%)	12 (31.6%)^*^p = 0.0452	8 (38%)^*^p = 0.0263
		20 (33.9%)^*^p = 0.0297	
Flares	0	8 (21%)^*^p = 0.0479	14 (66.7%)^*^p = 0.001 ^**^p = 0.0008
		22 (37.3%)^*^, *p* = 0.0020	
Hashimoto thyroiditis	0	17 (44.7%)^*^p = 0.0005	10 (47.6%)^*^p = 0.0008
		27 (45.8%)^*^p = 0.003	
Placental insufficiency	0	6 (15.8%) *p* = 0.1615	8 (38.1%) ^*^p = 0.0047
		14 (23.7%), ^*^p = 0.0308	
PE (%)	0	6 (15.8%) *p* = 0.1615	6 (28.6%)^*^p = 0.0241
		12 (20.3%), *p* = 0.0531	
PTB (%)	0	3 (7.9%) *p* = 1.000	2 (13.3%) *p* = 0.4922
		5 (8.5%), *p* = 0.5812	

*Conception after IVF and the presence of implantation failure (inability to achieve a clinical pregnancy after transfer of at least four good-quality embryos in a minimum of three fresh or frozen cycles) were investigated as signs of compromised fertility. Placental insufficiency, preeclampsia and preterm birth were used to evaluate pregnancy outcomes. Presence of flares, defined as the relapse of symptoms that can compromise the skin, the joints, or any other compromised organ and complement C3 consumption were used to evaluate disease activity*.

*N, number of patients; %, percentage of patients; IVF, in vitro fertilization; PE, preeclampsia; PTB, preterm birth*.

* *Different from patients with normal levels of vitamin D*.

** *Different from patients with deficient levels of vitamin D. P <0.05 is considered statistically significant*.

Around 50% of women with hypovitaminosis D conceived after IVF (57.1% in the deficient group and 39.5 in the insufficient group) and a higher incidence, though not statistically significant, of implantation failure was also observed in this group, suggesting an association between lower levels of vitamin D and compromised fertility in OAPS patients.

In accordance with previous studies, low levels of complement C3 were observed in 28% of all OAPS patients (45, 199, 200). Interestingly, 39.5% of the patients in the vitamin D deficient and 57.1% in the vitamin D insufficient presented low levels of C3, suggesting an association between lower levels of vitamin D and lower levels of C3, indicative of complement consumption/activation by autoantibodies. While lower C3 levels could be caused by complement C3 deficiency, none of these patients showed increased susceptibility to infection, characteristic of the rare, genetic C3 deficiency.

Monitoring serum levels of C3 to assess for disease activity is recommended in patients with autoimmune diseases, in particular APS. In this study, we found a correlation between decreased levels of C3 and flares in pregnant women with antiphospholipid antibodies. Thirty-seven percent of the OAPS patients with hypovitaminosis D showed disease flares in contrast to none in the group with normal vitamin D levels, emphasizing the link between hypovitaminosis D and immune dysregulation previously described.

Strikingly, autoimmune hypothyroidism (Hashimoto disease, HT) associated with anti-TPO and anti-Tg antibodies was detected in almost 50% of the patients with hypovitaminosis D (44.7% in the vitamin D deficient group and 47.6% of the vitamin D insufficient women). The 17 OAPS patients with normal vitamin D values were euthyroid. While several studies have shown a correlation between vitamin D deficiency and thyroid autoimmunity (84, 201). It is still unclear whether the hypovitaminosis D is the result of HT disease or a part of its cause. One patient in the vitamin D deficient group and one in the vitamin D insufficient group were also diagnosed with autoimmune disorder Sjogren syndrome.

Provocatively, knowing the role of the immune system, in placentation and placental development, the number of cases of placental insufficiency, determined by decreased uterine artery flow, was significantly higher in the OAPS women with hypovitaminosis D compared to vitamin D sufficient OAPS-women. A bigger number of PE cases was observed in the vitamin D insufficient group. Abnormal placentation and pregnancy complications such as PE were not observed in the OAPS patients with normal vitamin D values. 8.5% of OAPS women with hypovitaminosis D delivered preterm in contrast with 0% of the OAPS women with normal vitamin D levels. However, the difference did not reach statistical significance. There were no significant associations between lower levels of vitamin D and other variables such as age, parity and type of aPL autoantibodies.

In conclusion, hypovitaminosis D (<30 ng/mL) was documented in almost 80% of OAPS patients during the first trimester of pregnancy and was associated with complement activation, increased incidence of flares, presence of autoimmune thyroiditis, placental insufficiency, and a higher incidence of preeclampsia. If hypovitaminosis is the cause or the consequence of autoimmunity and adverse pregnancy outcomes needs to be addressed in further studies.

## Conclusions of the Observational Studies

While numerous clinical and experimental evidence suggest that vitamin D deficiency is an important factor in the pathogenesis of adverse pregnancy outcomes in APS and the proven immunomodulatory effects of vitamin D and its analogs, as previously described, the effects of vitamin D supplementation in the prevention and treatment of pregnancy disorders are not completely understood. Only small and non-controlled studies have been performed in humans; however, they seem to indicate there is a potential beneficial effect of vitamin D supplementation in modulating the immune system, preventing inflammation and protecting maternal and fetal health. Our small observational study suggests that subnormal vitamin D levels is another contributing factor to adverse pregnancy outcomes in women with APS. The cause-consequence effects and the risks and benefits of vitamin D supplementation in autoimmunity, in particular APS and HT, and high-risk pregnancies needs to be further investigated.

## Data Availability Statement

The datasets generated for this study are available on request to the corresponding author.

## Author Contributions

FC and GG reviewed the literature, interpreted the observational studies, performed statistical analysis, and wrote the review article. EL and KV were responsible for the supervision of the patients and data collection. GG created graphs.

### Conflict of Interest

The authors declare that the research was conducted in the absence of any commercial or financial relationships that could be construed as a potential conflict of interest.
